# Impact of the Stringent Stress Response on the Expression of Methicillin Resistance in *Staphylococcaceae* Strains Carrying *mecA*, *mecA1* and *mecC*

**DOI:** 10.3390/antibiotics11020255

**Published:** 2022-02-16

**Authors:** Catarina Milheiriço, Alexander Tomasz, Hermínia de Lencastre

**Affiliations:** 1 Laboratory of Molecular Genetics, Instituto de Tecnologia Química e Biológica António Xavier (ITQB NOVA), Universidade Nova de Lisboa, 2780-157 Oeiras, Portugal; lencash@rockefeller.edu; 2 Laboratory of Microbiology & Infectious Diseases, The Rockefeller University, New York, NY 10065, USA; tomasz@rockefeller.edu

**Keywords:** MRSA, methicillin genetic determinants, guanine metabolism

## Abstract

The acquisition of the resistance determinant *mecA* by *Staphylococcus aureus* is of major clinical importance, since it confers a resistant phenotype to virtually the entire large family of structurally diverse β-lactam antibiotics. While the common resistance determinant *mecA* is essential, the optimal expression of the resistance phenotype also requires additional factors. Previous studies showed that the great majority of clinical isolates of methicillin-resistant *S. aureus* (MRSA) have a heterogeneous resistant phenotype, and we observed that strains carrying methicillin genetic determinants other than *mecA* also produce similar heterogeneous phenotypes. All these strains were able to express high and homogeneous levels of oxacillin resistance when sub-inhibitory concentrations of mupirocin, an effector of the stringent stress response, were added to growth media. Our studies show that the gene *gmk*, involved in guanine metabolism, was one of the first genes to exhibit mutations in homoresistant (H*R) derivatives obtained through serial passages (with increasing concentrations of oxacillin) of the prototype *mecC*-carrying MRSA strain LGA251. All these observations led us to propose that a common molecular mechanism for the establishment of high and homogeneous oxacillin resistance must be present among isolates carrying different methicillin resistance determinants. In this work, we tested this hypothesis using whole-genome sequencing (WGS) to compare isogenic populations differing only in their degrees of oxacillin resistance and carrying various methicillin genetic determinants

## 1. Introduction

Infections caused by methicillin-resistant staphylococci are a significant burden on health-care systems [1]. Resistance to β-lactam antibiotics in *Staphylococcus aureus* is mediated by an alternative penicillin-binding protein with low affinity to virtually all β-lactam antibiotics encoded by *mecA* or by its homologues *mecB* or *mecC* [2,3,4,5]. Most strains carry the methicillin resistance determinant *mecA* gene as part of a foreign mobile genetic element—the Staphylococcal Cassette Chromosome *mec* (SCC*mec*) [6]. As with *mecA*, *mecC* is also located in an SCC*mec* element at the 3′ region of *orfX*, and, although being first identified in *S. aureus* strains of human and animal origins [7,8], it was also found in other staphylococcal species [9]. The *mecB* gene has been reported mostly in *Macrococcus,* either chromosomally or in a plasmid [10,11]. Recently, it was also detected on a plasmid in an *S. aureus* isolate [3].

Searching for the origin of the methicillin-resistant determinant in closely related species led to the identification of a close *mecA* homologue—*mecA1*—in *Mammaliicoccus sciuri* (formerly identified as *Staphylococcus sciuri* [12])*,* which was present in all the epidemiologically unrelated isolates tested [13], leading to the proposal of this homologue as the evolutionary precursor of the resistance determinant *mecA* carried by all methicillin-resistant *S. aureus* (MRSA) strains. Although the overwhelming majority of *M. sciuri* isolates that were initially examined were fully susceptible to β-lactam antibiotics [13], *M. sciuri* isolates from human clinical specimens were later reported with increased MICs for β-lactam antibiotics [14]. 

Interestingly, although antibiotic resistance is believed to arise primarily from human antibiotic overuse [15], a recent study [16] making use of time-resolved phylogenies suggested that particular lineages of *mecC*-MRSA appeared in European hedgehogs 200 years ago, i.e., more than a century before the first description of MRSA in humans. These lineages emerged in hedgehogs colonized with *S. aureus* and co-infected with a β-lactam-producer dermatophyte, *Trichophyton erinacei*. The *mecC*-MRSA lineages developed to resist the penicillin produced by *T. erinaceid* and eventually spread within local hedgehog populations and between hedgehogs and secondary hosts, such as livestock and humans [16]. 

The *mecA* gene is widely disseminated among staphylococcal species, and its expression is essential for the methicillin-resistant phenotype. However, the mechanism involved in the strain-to-strain variation of the phenotypic expression of methicillin resistance is still not well understood. Most MRSA strains exhibit a heterogeneous phenotype of β-lactam resistance under routine growth conditions: the majority of cells exhibiting variable but typically low-level resistance coexist with a few cells displaying homogeneous and high-level oxacillin resistance (H*R) [17,18]. This phenotype may have considerable clinical relevance since, upon being challenged by β-lactam agents, a homogeneous and highly oxacillin-resistant subpopulation may rapidly be selected. 

Several factors are known to contribute to β-lactam resistant phenotype. Studies with MRSA strain COL have shown that the optimal expression of the resistance phenotype requires not only the presence of *mecA* but also a large number of key additional chromosomal determinants—“auxiliary genes”, identified by Tn*551* mutagenesis [19,20]. TCA cycle has also been implicated in β-lactam H*R phenotype [21,22]. Environmental growth conditions are also known to affect the level of resistance and to be linked with stress response stages in other bacterial species, due to alterations in the levels of (p)ppGpp molecules under those conditions [23]. In 2013, it was proven that *relA* (coding for (p)ppGpp synthesis, an effector of the stringent stress response) was involved in the oxacillin H*R phenotype of an MRSA laboratory model strain [24]. More recently, Dr. Hiramatsu’s group found a new gene—*ehoM*—with an essential role in high-level β-lactam resistance in MRSA via the stringent response [25]. Additionally, several genetic determinants belonging to diverse functional categories including guanine metabolism were identified as being involved in the heterogeneous expression of oxacillin resistance in four historically early MRSA strains and in the major contemporary MRSA clone, USA300 [17,18]. 

In previous studies, we showed that the gene *gmk*, coding a guanylate kinase, was mutated in H*R step-selection derivatives obtained through serial passages of the prototype *mecC*-carrying MRSA strain LGA251 with increasing concentrations of oxacillin [26]. We have also noticed that oxacillin heteroresistant phenotypes in LGA251, as well as in SS37, a *M. sciuri* strain carrying *mecA1*, could be altered to a H*R phenotype if sub-inhibitory concentrations of mupirocin were added to the growth medium [26,27]. This led us to hypothesize that a common molecular mechanism for the establishment of high and homogeneous levels of oxacillin resistance involving a stringent stress response could be present among these closely related species regardless of the methicillin genetic determinant carried. To test our hypothesis, we compared isogenic strains, differing only in their degrees of oxacillin resistance, recovered from three heteroresistant parental populations carrying different methicillin genetic determinants, using whole-genome sequencing (WGS), in an attempt to identify the genes that may be responsible for the H*R phenotypes. 

## 2. Results

### 2.1. Selection of Homogenous Highly Resistant (H*R) Subpopulations

The procedure used for the isolation of the H*R derivatives was adapted from [17]. First, 30 H*R isolates, 10 for each parental strain, were picked from TSA plates supplemented with 3, 50, or 200 μg/mL oxacillin (for LGA251, RUSA239, and SS37, respectively). Colonies were stabilized by four passages on TSA in the absence of oxacillin. Then, 15 H*R, 5 for each parental strain, were selected based on their homogeneous and high-level oxacillin resistance phenotypes, as depicted by population analysis performed. 

**Figure 1:**
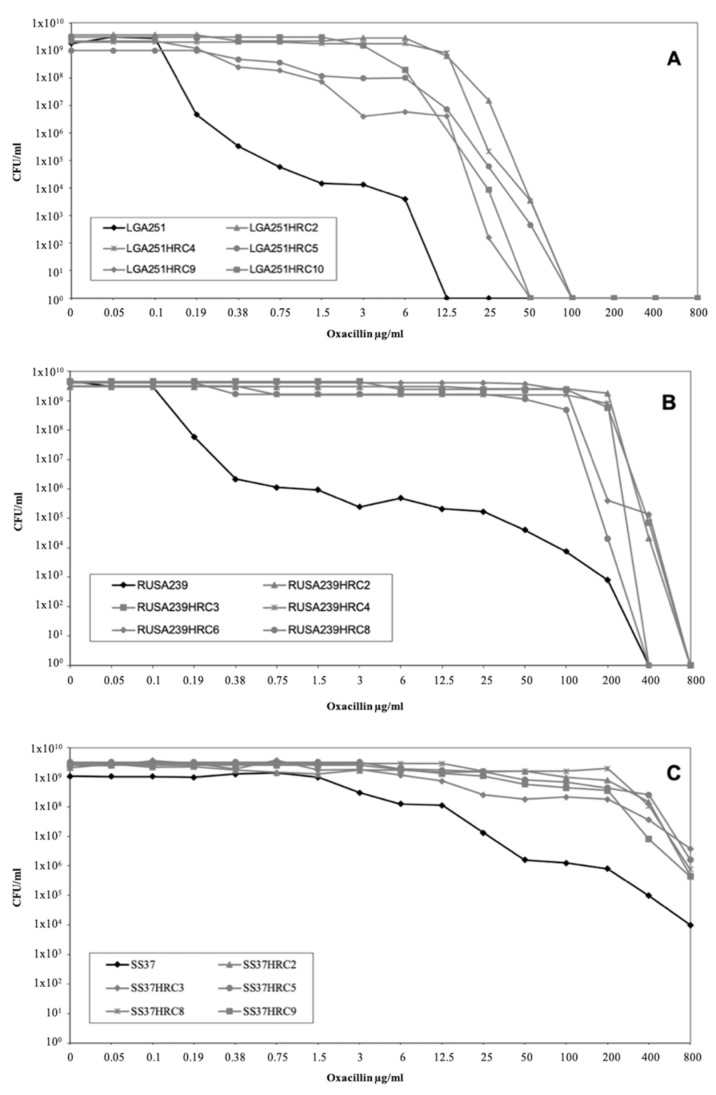
Population analysis profiles showing the different profiles between (**A**) LGA251, (**B**) RUSA239, and (**C**) SS37 parental heteroresistant populations (black lines) and the respective homoresistant (H*R) sub-populations (grey lines).

The population analysis profiles (PAPs) for the parental strains LGA251, RUSA239, and SS37 and for the selected H*R derivatives show the establishment of a high and homogeneous oxacillin resistance phenotype among the H*R isolates in contrast with the heterogeneous phenotype of the parental strains (Figure 1).

### 2.2. Whole-Genome Sequencing of Parental Strains and Their Subpopulations

Fifteen H*R derivatives with high and homogeneous levels of oxacillin resistance were selected to perform WGS. The genomes of the “parental” populations of RUSA239 and SS37 were also sequenced to use as reference genomes in bioinformatic analysis. Two different approaches have been followed to compare the genomes of the H*R derivatives with the ones of their parental strains. For LGA251 and its H*R derivates, we performed a remapping approach using the published genome of LGA251 (NC_017349.1) as a reference and mapping against it the LGA251 H*R derivatives’ quality processed reads retrieved from the Trimmomatic program after performing multisoftware pipeline INNUca. For RUSA239 and SS37, SPAdes de novo assemblies of the parental strains retrieved through the INNUca pipeline were used as references against which the respective H*R derivatives’ quality processed reads were mapped. 

### 2.3. Affected Genes and Their Functional Category

Each H*R subpopulation showed one to three mutations compared to the parental population, with the exception of SS37HRC3, which carried 10 mutations, and RUSA239HRC8, for which we could not detect mutations in raw reads (Table 1). 

In LGA251 H*R derivatives, two loci were repeatedly affected: *hpt* in LGA251HRC2 and LGA251HRC5, encoding a hypoxanthine phosphoribosyltransferase that has been suggested to be involved in guanine metabolism in several Firmicutes [28,29,30]; and tRNA_48 in LGA251HRC4 and LGA251HRC10, involved in protein biosynthesis. Curiously, LGA251HRC4 and LGA251HRC10 showed exactly the same mutation in tRNA_48, a single nucleotide polymorphism (SNP) at nucleotide 1934923 leading to the substitution of a guanine by an adenine at the 3′ end of this tRNA. LGA251HRC2 carries, besides the mutation in *hpt,* an additional mutation in a putative pathogenicity island. LGA251HRC9 carries two SNP mutations, one in a putative membrane protein and one in *mutL*, coding for the DNA mismatch repair protein MutL [31].

No common mutations have been found among RUSA239 H*R derivatives. The four derivatives each showed a single SNP: RUSA239HRC3 and RUSA239HRC6 in the same intergenic region; RUSA239HRC2 in *pheS,* coding for the phenylalanyl-tRNA synthase involved in protein biosynthesis; and RUSA239HRC4 in *relA2,* a GTP pyrophosphokinase involved in the synthesis of (p)ppGpp, the stringent stress response regulator molecule. 

*guaB,* which codes for the IMP dehydrogenase involved in guanine metabolism [32], was repeatedly affected in all SS37 H*R derivatives, except in SS37HRC3. *guaB* was the unique mutation found in SS37HRC9, and in SS37HRC8 an additional mutation was found in an intergenic region. SS37HRC5 showed, besides the mutation in *guaB*, an SNP mutation in *ftnA,* coding for ferritin, an iron storage protein [33]. SS37HRC2 showed two additional mutations in two SNPs: one in *tagF_2*, coding for a glycosyl glycerol phosphate transferase involved in teichoic acid biosynthesis [34], and the other in *ykfC,* coding for a peptidoglycan endopeptidase of the NlpC/P60 family of proteins [35].

Of all the isogenic derivative strains analyzed, SS37HRC3 was the only one that presented more than three mutations affecting different functional categories (see Table 1 for details) that may be explained by the presence of an SNP mutation in the *mutL* gene coding for a DNA mismatch repair protein [31]. 

## 3. Discussion

**Figure 2:**
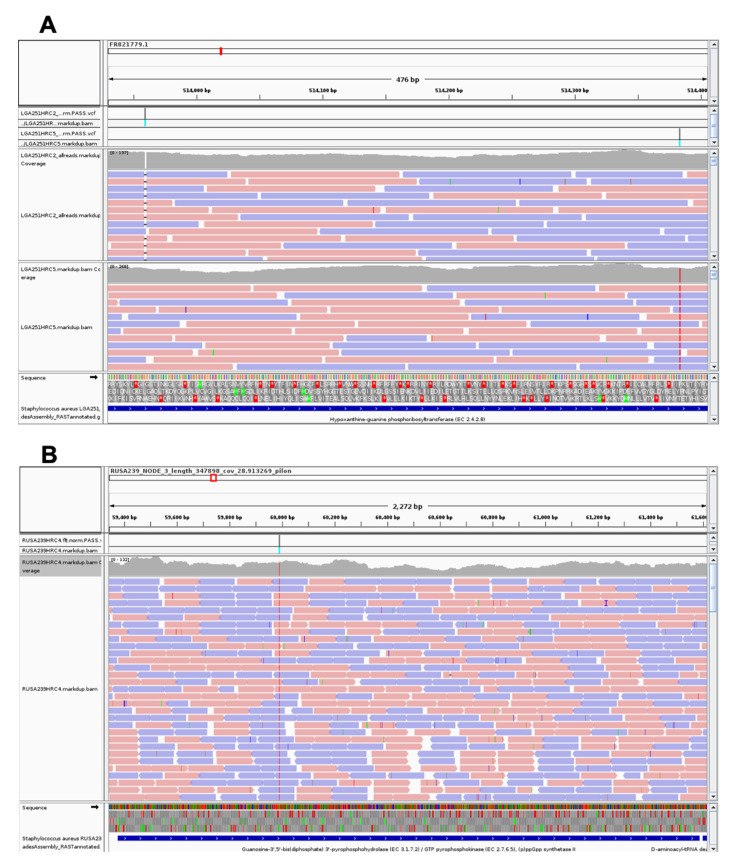

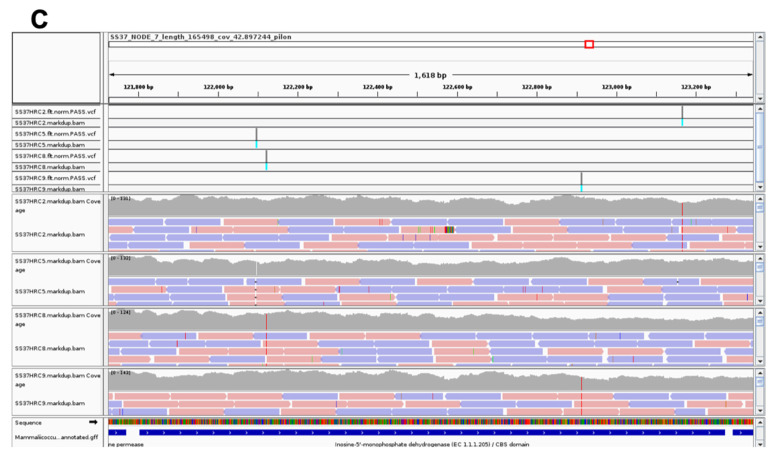
Overview of the mutations found in genes related to guanine metabolism: (**A**) *hpt* in LGA251HRC2 and LGA251HRC5; (**B**) *relA2* in RUSA239HRC4; (**C**) *guaB* in SS37HRC2, SS37HRC5, SS37HRC8, and SS37HRC9. Mutations are shown as grey bars.

**Figure 3:**
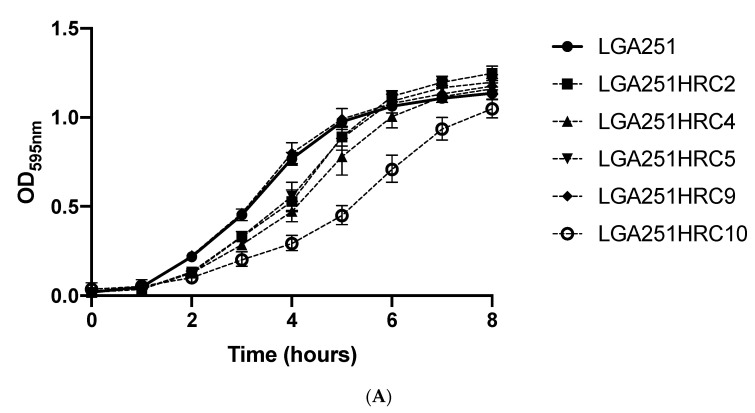

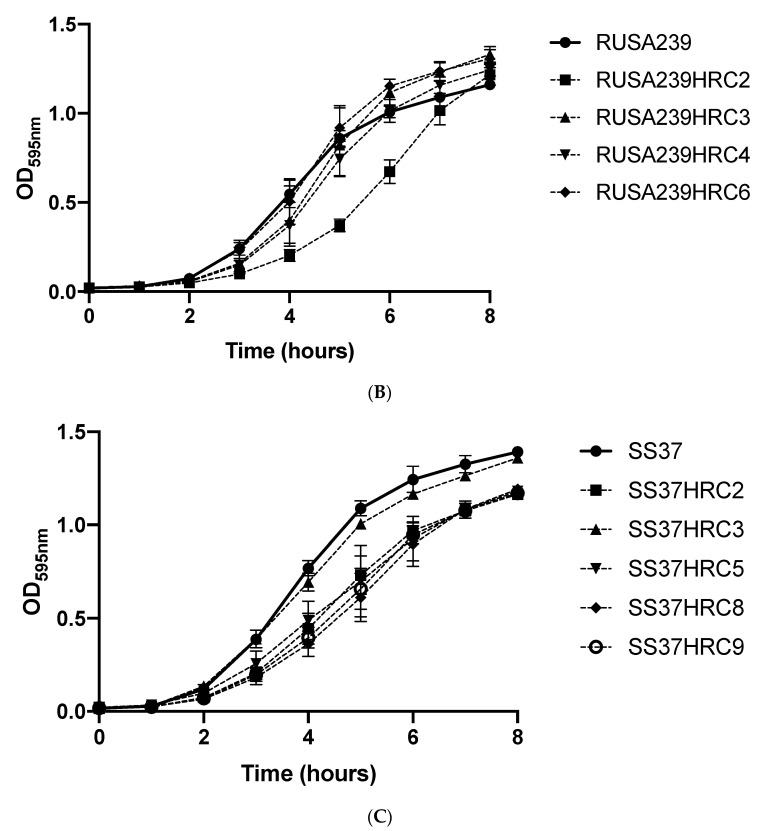
Growth curves of (**A**) LGA251, (**B**) RUSA239, and (**C**) SS37 parental heteroresistant populations and the respective H*R sub-populations. Bacterial cultures were grown in TSB at 37 °C with aeration. All points represent the geometric mean of three technical plus three biological triplicates. Error bars represent 95% confidence intervals.

Although no common mutated genes were found in the H*R derivatives (representing the three genetic backgrounds used in the study), in all of them, we were able to detect the presence of mutated genes related to guanine metabolism: 

– In LGA251 H*R derivatives, both mutations found in *hpt* (Figure 2) gave rise to truncated proteins: in LGA251HRC2, the frameshift mutation gave rise to a protein only 14% of the normal size (25 aa versus 179 aa), whereas in LGA251HRC5, the SNP mutation led to the formation of a premature STOP codon at the amino acid 164. Mutations in *hpt* were already described in MRSA populations with an H*R-associated phenotype belonging to different genetic backgrounds and carrying *mecA* as the methicillin genetic determinant [17,18]. The production of function-deficient Hpt proteins in these two LGA251 H*R derivatives will impact GTP availability in the cell due to its role in guanine metabolism, namely on the availability of GMP, which is then converted to GTP by the Gmk enzyme. Decreased intracellular levels of GTP is a phenomenon that also occurs when the stringent stress response is activated [36]. Low levels of GTP are thought to impair transcription and translation processes leading to slow growth and—as a consequence—to an increase in the tolerance of bacterial cells to antimicrobials [36], which may explain the H*R phenotype presented by strains LGA251HRC2 and LGA251HRC5 and their slower growth in comparison with the parental strain LGA251, as depicted in Figure 3 and Table S3. 

– In the derivative RUSA239HRC4, an SNP mutation in *relA2* has been detected (Figure 2), which gave rise to a premature STOP codon after the hydrolase domain of the encoded protein. The gene *relA2* encodes for a GTP pyrophosphokinase that promotes the synthesis of (p)ppGpp, an effector of the stringent stress response. Mutations in *relA2* have been linked with oxacillin H*R phenotypes in MRSA isolates [17,18,24]. However, in most cases, the synthetase domain of the enzyme remained active, and the H*R phenotypes have been associated with the synthesis of higher amounts of (p)ppGpp, which, in turn, would activate the stringent stress response. In contrast, the mutated *relA2* found in RUSA239HRC4 produces a smaller enzyme lacking the synthetase and regulatory domains (TGS and ACT). Another MRSA isolate showing an oxacillin H*R phenotype—BB8, described by Dordel et al. [17]—was characterized by the presence of a mutation that impaired the synthetase domain of RelA due to a premature STOP codon at the beginning of this domain. Intriguingly, in both cases, those mutations were the only ones obtained in the WGS analysis performed, which could indicate that these isolates may have additional undetected mutations. This may be due to the sequencing technology used (short-reads) or the minimum criteria defined to call SNPs in both studies. Additional studies including the use of long-reads sequencing technology are needed to better understand the mechanism responsible for the H*R phenotype in these two isolates.

– All except one SS37 H*R derivatives showed the presence of a mutation in the *guaB* gene (Figure 2). Despite the different nature of the mutations found in *guaB*, which were scattered all over the gene, all of them led to the formation of truncated proteins due to the presence of premature STOP codons in the new coding sequences. *guaB* codes for IMP dehydrogenase, which catalyzes the conversion of inosine 5’-phosphate (IMP) to xanthosine 5’-phosphate (XMP), an essential step in the de novo synthesis of guanine nucleotides [32]; therefore, it plays an important role in the regulation of cell growth, as depicted in Figure 3 and Table S3, which could explain the H*R phenotype seen in these H*R derivatives. 

Of further note are other mutations that are not involved in guanine metabolism but may potentially influence the H*R phenotype: 

– LGA251HRC4 and LGA251HRC10 each carry the same single SNP mutation at nucleotide 1934923, leading to the substitution of a guanine for an adenine in the acceptor stem of a tRNA annotated as tRNA_48 in the LGA251 genome and as tRNA-Met in several *S. aureus* genomes. This mutation is close to the CCA 3′-terminal group used to attach amino acids to tRNAs, which can impair protein translation and consequently lead to slow growth. In fact, these two H*R derivatives showed a slower growth in comparison with their parental strain, as illustrated in Figure 3 and in Table S3. Since β-lactam antibiotics are effective on actively growing cells, slower growing cells would benefit under these conditions and would support higher concentrations of the antibiotic than when actively growing. Although these two derivatives share the same SNP, their growth is somewhat different, as seen in Figure 3. As this study was performed with short-read sequencing technology, and the genomes were not closed, one of these derivatives may have additional mutations that could not be detected here with the criteria used to call SNPs, which may eventually explain this difference.

– RUSA239HRC2 carries an SNP mutation that led to the substitution of phenylalanine residue by a serine at amino acid 254 of PheS—the alpha subunit of the phenylalanyl-tRNA synthase, a residue that has different chemical properties. Phe254 is part of the large hydrophobic pocket that forms the active site of this essential enzyme for protein biosynthesis [37]. Thus, mutations in this location can influence cellular growth. Indeed, RUSA239HRC2 clearly showed slower growth in comparison with the parental strain RUSA239 (Figure 3 and Table S3).

RUSA239HRC3 and RUSA239HRC6 showed a unique SNP in the same intergenic region, around 300 bp upstream of *glmS* gene, coding for a glutamine-fructose-6-phosphate aminotransferase (isomerizing) that Komatsuzawa et al. proposed to be involved in glucosamine-6-P formation from fructose-6-P to be then incorporated in peptidoglycan biosynthesis [38]. Although being near the 5′ end of the *glmS*, the mutations found in RUSA239HRC3 and RUSA239HRC6 are not present in typical locations for regulatory processes. Moreover, the growth differences of these two derivatives in comparison to the reference strain were not statistically significant (Table S3), pointing to a need for further studies to ascertain the importance of these unique mutations in the expression of this gene and in the H*R phenotypes of these strains.

In conclusion, we could not detect mutations affecting the same genetic determinants in the 15 oxacillin H*R derivative populations in this study, which were isolated from three parental strains carrying different methicillin resistance determinants—*mecA*, *mecC,* and *mecA1*. However, we did find mutations in three genetic determinants involved in guanine metabolism (and consequently in the stringent stress response in derivatives of the three parental populations). These genetic determinants—*hpt*, *relA2*, and *guaB*—were previously reported as implicated in H*R phenotypes in MRSA subpopulations belonging to different genetic backgrounds, strengthening the evidence for the involvement of the stringent stress response in defining the antibiotic resistance level, regardless of clonal type or the methicillin genetic determinant the strains carried. Additional mutations that may impair protein biosynthesis have also been detected in some derivatives, a phenomenon also known to occur when the stringent stress response is activated.

## 4. Materials and Methods

### 4.1. Bacterial Strains, Media, and Growth Conditions

Three parental populations carrying different methicillin genetic determinants were used in this study: (i) LGA251 [7], an *S. aureus* veterinary strain carrying the *mecC* gene as the methicillin genetic determinant; (ii) RUSA239 [19], an *S. aureus* auxiliary mutant of strain COL carrying *mecA*; and (iii) SS37 [14], an *M. sciuri* clinical isolate carrying *mecA1*, a precursor of the *mecA* gene. Strains were grown in tryptic soy broth (Difco Laboratories, Sparks, MD, USA) or on tryptic soy agar (Difco Laboratories) at 37 °C with aeration.

### 4.2. Antibiotic Susceptibility

The preliminary detection of heteroresistance in the parental strains was performed using Etests (bioMérieux, Marcy-l’Étoile, France) and then confirmed by population analysis profiles (PAPs). The Etest was conducted by spreading a small aliquot of an overnight culture diluted to an optical density at 620 nm (OD620) of 0.08 onto TSA plates and adding an oxacillin Etest strip onto the surface of the plates. After 24 h of incubation at 35 °C, the plates were inspected for the presence of heteroresistant subpopulations growing within the inhibition zone of the antibiotic. PAP testing was performed as previously described [17]. In summary, overnight cultures were plated on TSA with increasing concentrations (2-fold) of oxacillin, and the oxacillin MICs were determined after 48 h of incubation at 37 °C. Heteroresistance was confirmed by visual inspection of the PAPs. Selected sub-populations whose main population reached the clinical resistance level (4 μg/mL for *S. aureus* and 0.5 μg/mL for *M. sciuri*) and that had a MIC at least 16-fold higher than the highest concentration of the drug that does not affect growth of the main susceptible parental population were considered high oxacillin resistant derivatives (Table S1). MIC was considered the antibiotic concentration that inhibited the growth of 99.9% of the cells.

### 4.3. Selection of Homogenous Highly Resistant Subpopulations

An adaptation of the protocol of Dordel et al. was used for the selection of the H*R isolates [17]. Briefly, 10 H*R isolates per parental strain in this study have been picked from TSA plates supplemented with 3, 50, or 200 μg/mL oxacillin for LGA251, RUSA239, and SS37, respectively. Oxacillin concentrations used for the isolation of the H*R were chosen, taking into account the PAP results of the parental strains. Next, the H*R isolates were passaged four times onto fresh TSA plates, after which the isolates were tested for resistance level by Etest and population analysis. A total of 15 H*R isolates (5 H*R isolates per parental strain) with high and homogeneous levels of oxacillin resistance were selected to perform whole-genome sequencing. 

### 4.4. Whole-Genome Sequencing

Genomic DNA was extracted from both the parental strains (RUSA239 and SS37) and the 15 isolated H*R strains using the Qiagen DNeasy blood and tissue kit (Qiagen, Ambion Inc., Austin, TX, USA). Paired-ended (2 × 150 bp) sequencing was performed at the Instituto Gulbenkian de Ciência ([IGC] Oeiras, Portugal) using the Illumina NextSeq500 platform. The quality control of reads, de novo assembly, contigs quality assessment, and possible contamination search were carried out using the multisoftware pipeline INNUca version 3.1 (https://github.com/B-UMMI/INNUca, accessed on 14 February 2022) [39]. The mean depth of coverage (after quality control) ranged from 92- to 186-fold. The genome of LGA251 (available at NCBI under the accession number NC_017349.1) was used as the reference for the LGA251 H*R derivatives study. For the RUSA239 and SS37 studies, we used as reference genomes de novo assemblies obtained with SPAdes run in the INNUca pipeline. The quality processed reads of each H*R strain were mapped against the respective “parental” strain assemblies using bwa-mem (version 0.7.17) [40], and variant calling was performed using bcftools (version 1.8) [41], with the following criteria: (i) a minimum mapping quality of 30; (ii) a minimum number of 25 high-quality processed reads for the alternate allele; and (iii) a minimum proportion of 80% of quality processed reads at the alternate allele differing from the reference. All mutations were inspected and confirmed using Integrative Genomics Viewer (http://software.broadinstitute.org/software/igv/, accessed on 14 February 2022) [42]. Selected assemblies for parental isolates were inspected and subsequently annotated using Prokka (https://github.com/tseemann/prokka, accessed on 14 February 2022) (version 1.13.3) [43] and PATRIC RASTtk-enabled Genome Annotation Service [44]. Table S2 shows the quality assessment statistics for the assemblies generated from the sequenced genomes.

### 4.5. Accession Number(s)

The raw reads used in this study have been deposited in the European Nucleotide Archive (ENA) under BioProject nr. PRJEB49801. The accession numbers for each are provided in Table S2.

### 4.6. Growth Curves

Overnight cultures from parental and H*R strains were diluted to an optical density (OD_620_) of 0.05 into fresh TSB and incubated at 37 °C with shaking (180 rpm) during an 8 h period in the Infinite F200 PRO Tecan microplate reader. The optical density was monitored at 595 nm every 60 min. Results were plotted as the geometric mean of three technical plus three biological triplicates with 95% confidence intervals using the software GraphPad Prism version 8.4.3 (GraphPad Software, San Diego, CA, USA). Statistical analysis was performed using the Mann–Whitney test (Table S3). A *p*-value lower than 0.05 was considered significant.

## Figures and Tables

**Table 1:** Mutated loci identified in high oxacillin resistant (H*R) isolates carrying different methicillin genetic determinants.

H*R Isolate	Nucleotide Change (5′ → 3′) ^a^	Amino Acid Change	Affected Genes	Locus Tag(s) in Reference Strains ^b^	Product ^c^
LGA251HRC2	66delG	Gly23fs	*hpt*	SARLGA251_04440	putative hypoxanthine phosphoribosyltransferase
	857450insA				putative pathogenicity island
LGA251HRC4	1934923G → A		*tRNA_48*		tRNA-Met-CAT
LGA251HRC5	490C → T	Arg164 *	*hpt*	SARLGA251_04440	putative hypoxanthine phosphoribosyltransferase
LGA251HRC9	297A → G	Ile99Met		SARLGA251_00130	putative membrane protein
	1021delA	Asn343fs	*mutL*	SARLGA251_12070	DNA mismatch repair protein MutL
LGA251HRC10	1934923G → A		*tRNA_48*		tRNA-Met-CAT
RUSA239HRC2	761T → C	Phe254Ser	*pheS*	SACOL1148	phenylalanyl-tRNA synthetase, alpha subunit
RUSA239HRC3	2208425C → T				Intergenic region
RUSA239HRC4	595C → T	Gln199 *	*relA2*	SACOL1689	GTP pyrophosphokinase
RUSA239HRC6	2208422C → A				Intergenic region
SS37HRC2	136A → T	Leu46Met	*tagF_2*	NCTC12103_02001	glycosyl glycerol phosphate transferase involved in teichoic acid biosynthesis
	1363C → T	Gln455 *	*guaB*	NCTC12103_02801	IMP dehydrogenase/CBS domain
	657delC	Ile219fs	*ykfC*	NCTC12103_02764	L-alanyl-gama-D-glutamyl-L-diamino acid endopeptidase—NLPC/P60 family protein
SS37HRC3	881C → T	Gly294Asp	*ptsI*	NCTC12103_01951	phosphoenolpyruvate-protein phosphotransferase of PTS system
	478delT	Met160Trp		NCTC12103_01955	radical activating enzyme protein
	419G → A	Ala140Val	*purM*	NCTC12103_01966	phosphoribosylformylglycinamidine cyclo-ligase
	227C → T	Gly76Glu	*eamB_2*	NCTC12103_02032	amino acid transporter LysE
	1065A → G		*oppA_1*	NCTC12103_02094	oligopeptide ABC transporter periplasmic oligopeptide-binding protein
	409insT	Gln137Thr	*mutL*	NCTC12103_01639	DNA mismatch repair protein
	517delA	Lys173fs	*lutA*	NCTC12103_00751	glycolate oxidase iron-sulfur subunit
	646delT	Thr216Leu		NCTC12103_01160	Gas vesicle protein
	353G → A	Ala118Val	*sftA*	NCTC12103_01157	cell division protein DNA translocase FtsK
	129C → T		*licB_2*	NCTC12103_02427	phosphotransferase system cellobiose-specific component IIB
SS37HRC5	294delC	Asn98fs	*guaB*	NCTC12103_02801	IMP dehydrogenase/CBS domain
	331C → T	Glu111Lys	*ftnA*	NCTC12103_00955	Bacterial non-heme Ferritin
SS37HRC8	400274delT				Intergenic region
	319C → T	Gln107 *	*guaB*	NCTC12103_02801	IMP dehydrogenase/CBS domain
SS37HRC9	1111G → T	Glu371 *	*guaB*	NCTC12103_02801	IMP dehydrogenase/CBS domain

^a^ The nucleotide positions in intergenic regions refer to the nucleotide positions in the reference genomes of strains LGA251, COL, and NCTC1203; ^b^ according to the genome annotations of the reference strains LGA251 (accession number NC_017349.1), COL (accession number CP000046.1), and NCTC12103 (accession number LS483305.1); ^c^ according to Prokka and RASTtk annotation services; *—STOP codon; fs—frameshift; ins—insertion; del—deletion.

## Data Availability

The data presented in this study are available in supplementary material. Raw read data is deposited in the European Nucleotide Archive (ENA) under BioProject nr. PRJEB49801.

## Supplementary Materials

The following supporting information can be downloaded at: https://www.mdpi.com/article/10.3390/antibiotics11020255/s1, Table S1. H*R derivatives MIC and Fold change determination. Table S2. Quality assessment statistics for the assemblies generated from the sequenced genomes. Data provided by INNUCA’s samples reports. Table S3. Mann-Whitney test analysis performed to detect statistical significant differences in growth curves of the H*R derivatives in relation to the parental strains at early exponential phase of growth time points (at 3 and at 4 h).
