# Supplementary material for: Impact of the Stringent Stress Response on the Expression of Methicillin Resistance in Staphylococcaceae Strains Carrying mecA, mecA1 and mecC
Source: Antibiotics (Basel). 2022 Feb 16;11(2):255. doi: 10.3390/antibiotics11020255 (PMC8868139; doi:10.3390/antibiotics11020255)
Supplement: Supplementary file 1 [file antibiotics-11-00255-s001.zip › antibiotics-1562715-supplementary.pdf]

Table S1. H*R derivatives MIC and Fold change determination.

Isolate	Highest concentration of oxacillin that does not affect growth of the main susceptible parental population (µg/ml)	MIC (µg/ml)	Fold change*
LGA251	0.1		
LGA251HRC2		50	18
LGA251HRC4		25	16
LGA251HRC5		25	16
LGA251HRC9		25	16
LGA251HRC10		25	16
RUSA239	0.1		
RUSA239HRC2		400	24
RUSA239HRC3		400	24
RUSA239HRC4		400	24
RUSA239HRC6		200	22
RUSA239HRC8		200	22
SS37	1.5		
SS37HRC2		800	18
SS37HRC3		> 800	> 18
SS37HRC5		800	18
SS37HRC8		800	18
SS37HRC9		800	18

* Fold change between the H*R derivative MIC and the highest concentration of oxacillin that does not affect growth of the main susceptible parental population.

Table S2. Quality assessment statistics for the assemblies generated from the sequenced genomes. Data provided by INNUCA's samples reports.

Isolate	Raw reads accession number	Number of contigs	Coverage	Assemblies Total length (bp)	Percentage of completion *	N50	L50
LGA251HRC2	ERR7959771	29	134.04	2717791	98.8	301466	4
LGA251HRC4	ERR7959772	28	126.51	2717110	98.8	301466	4
LGA251HRC5	ERR7959773	35	186.17	2718628	98.8	301466	4
LGA251HRC9	ERR7959774	32	109.43	2717905	98.8	301466	4
LGA251HRC10	ERR7959775	30	126.63	2717078	98.8	301466	4
RUSA239	ERR7959776	39	99.63	2780349	99	347898	3
RUSA239HRC2	ERR7959777	40	106.27	2779620	99	231693	5
RUSA239HRC3	ERR7959778	36	114.13	2780561	99	394410	3
RUSA239HRC4	ERR7959779	32	111.27	2780840	99	577863	2
RUSA239HRC6	ERR7959780	34	123.81	2782760	99.1	292725	4
RUSA239HRC8	ERR7959781	38	128.44	2781630	99	394410	3
SS37	ERR7959782	66	93.78	2912294	103.6	175761	6
SS37HRC2	ERR7959783	65	113.11	2912065	103.6	166665	7
SS37HRC3	ERR7959784	69	131.02	2913194	103.6	216617	6
SS37HRC5	ERR7959785	56	103.95	2907183	103.4	175756	6
SS37HRC8	ERR7959786	58	92.44	2905912	103.3	174955	6
SS37HRC9	ERR7959787	56	113.16	2912791	103.6	222677	6

* LGA251 genome size (accession number NC_017349.1), COL genome size (accession number CP000046.1) and NCTC12103 genome size (accession number LS483305.1) were used to calculate the percentage of completion of LGA251H*R derivatives, RUSA239H*R derivatives and SS37H*R derivatives, respectively.

Table S3. Mann-Whitney test analysis performed to detect statistical significant differences in growth curves of the H*R derivatives in relation to the parental strains at early exponential phase of growth time points (at 3 and at 4h).

Reference strain	H*R derivative	Sum of ranks for the reference	Sum of ranks for the H*R derivative	Mann-Whitney U	Statistical Significant?
LGA251	LGA251HRC2	432	234	63	Yes
	LGA251HRC4	441	225	54	Yes
	LGA251HRC5	419	247	76	Yes
	LGA251HRC9	316	350	145	No
	LGA251HRC10	495	171	0	Yes
RUSA239	RUSA239HRC2	480	186	15	Yes
	RUSA239HRC3	377	289	118	No
	RUSA239HRC4	417	249	78	Yes
	RUSA239HRC6	348	318	147	No
SS37	SS37HRC2	439	227	56	Yes
	SS37HRC3	362	304	133	No
	SS37HRC5	434	232	61	Yes
	SS37HRC8	468	198	27	Yes
	SS37HRC9	453	213	42	Yes
